# Combined *In Silico* and *In Vivo* Analyses Reveal Role of *Hes1* in Taste Cell Differentiation

**DOI:** 10.1371/journal.pgen.1000443

**Published:** 2009-04-03

**Authors:** Masato S. Ota, Yoshiyuki Kaneko, Kaori Kondo, Soichi Ogishima, Hiroshi Tanaka, Kazuhiro Eto, Takashi Kondo

**Affiliations:** 1 Section of Molecular Craniofacial Embryology, Tokyo Medical and Dental University, Bunkyo-ku, Tokyo, Japan; 2 Kondo Research Unit, Neuro-Developmental Disorder Research Group, Brain Science Institute, Institute of Physical and Chemical Research (RIKEN), Wako, Saitama, Japan; 3 Department of Bioinformatics, Tokyo Medical and Dental University, Bunkyo-ku, Tokyo, Japan; University of Colorado Denver, United States of America

## Abstract

The sense of taste is of critical importance to animal survival. Although studies of taste signal transduction mechanisms have provided detailed information regarding taste receptor calcium signaling molecules (TRCSMs, required for sweet/bitter/umami taste signal transduction), the ontogeny of taste cells is still largely unknown. We used a novel approach to investigate the molecular regulation of taste system development in mice by combining *in silico* and *in vivo* analyses. After discovering that TRCSMs colocalized within developing circumvallate papillae (CVP), we used computational analysis of the upstream regulatory regions of TRCSMs to investigate the possibility of a common regulatory network for TRCSM transcription. Based on this analysis, we identified *Hes1* as a likely common regulatory factor, and examined its function *in vivo*. Expression profile analyses revealed that decreased expression of nuclear HES1 correlated with expression of type II taste cell markers. After stage E18, the CVP of *Hes1^−/^* ^−^ mutants displayed over 5-fold more TRCSM-immunoreactive cells than did the CVP of their wild-type littermates. Thus, according to our composite analyses, *Hes1* is likely to play a role in orchestrating taste cell differentiation in developing taste buds.

## Author Summary

The sensation of taste is composed of five basic modalities: sweet, bitter, umami, sour, and salty. Specialized taste cells perceive the various chemical cues within food. About 100 taste cells assemble into onion-shaped clusters called taste buds, which are located on taste papillae in the tongue epithelium and on oral mucosa. Of the five taste modalities, the taste stimulants responsible for sweet, bitter, and umami tastes are recognized by a group of G protein–coupled taste receptors, and the signal transduction pathways utilized following receptor stimulation share common molecules. However, it is still largely unknown how these molecules are regulated during taste cell development. We performed computer analyses based on previously known information about signal transduction pathways involved in the taste-sensing system to identify taste stem cells/progenitor factors of type II taste cells (responsible for sweet, bitter, and umami taste sensations). We found several transcription factors likely to bind to the regulatory regions of taste-related calcium signaling molecules (TRCSMs), and identified *Hes1* as a potential candidate for common regulatory factors of TRCSMs. *In vivo* analyses using wild-type and *Hes1* mutant mice confirmed that *Hes1* regulates differentiation of bitter-, sweet-, and umami-sensing cells.

## Introduction

Taste is one of the major chemosensory systems enabling animals to perceive crucial environmental stimuli. It performs the vital role of helping animals to identify favorable nutrition sources, as well as to avoid toxic substances, making taste a fundamental sensory recognition system that is required for survival [1],[2]. While the ontogeny of the other special sense organs has been studied in depth at a molecular level [3]–[5], the development of taste remains to be clarified.

Taste buds are the sensory end organs for gustation, and are located on the epithelium of the tongue and palate. On the tongue, they reside on three types of papillae, i.e., fungiform, foliate, and circumvallate [2],[6],[7]. In adult mammals, each taste bud comprises groups of 50–100 spindle-shaped epithelial cells and a small number of proliferative cells [8],[9]. Taste bud cells are heterogeneous in terms of gene expression profiling of individual taste cells, as well as in their ultrastructural characteristics. [8], [10]–[14]. Ultrastructual studies have revealed three distinct anatomical types of spindle-shaped epithelial cells within each taste bud: type I (dark), type II (light), and type III (intermediate) cells [8],[10],[11]. Type II cells have a characteristic large round nucleus and are responsible for the sweet, bitter, and umami taste sensations [2], [6]–[8],[10]. These cells express a number of G protein–coupled receptors and common downstream transduction components called taste receptor calcium signaling molecules (TRCSMs; e.g., PLCβ2, gustducin [GNAT3], and IP3R3 [ITPR3]) [2],[6],[7],[10]. Although several studies have examined the lineage of taste cells [8],[10],[15],[16], the molecular mechanisms of cell differentiation in developing taste buds have remained elusive. We took a novel approach toward investigating taste cell development in mice by combining *in silico* and *in vivo* analyses of the TRCSM transcription regulatory network in type II taste cells.

## Results

### Early Phase of TRCSM Expression

**Figure 1:**
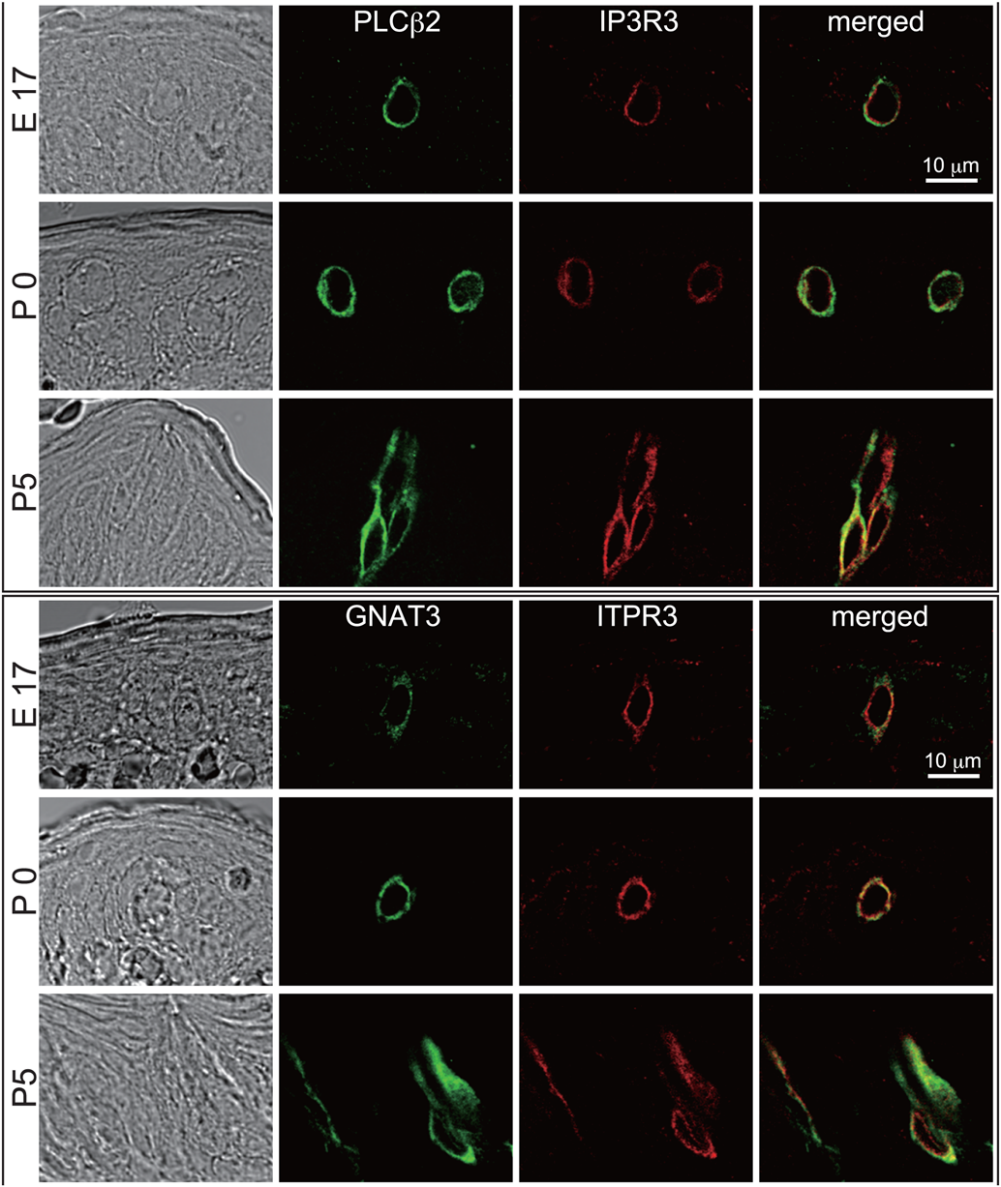
Colocalization of TRCSMs in early developing CVP. Immunohistochemical analyses of PLCβ2 (green signal) and IP3R3 or gustducin (red signal) in developing taste buds in CVP from stages E17 to P5. PLCβ2 and IP3R3 or gustducin colocalize within the same cells, at least until P5. Scale bar, 10 µm.

We examined the expression of TRCSMs in the epithelium of presumptive circumvallate papillae (CVP) during mouse embryogenesis. The papilla structure of CVP is already visible before embryonic day 14 (E14) [17]. We examined expression of five TRCSMs—PLCβ2, gustducin, IP3R3, Ggamma13 (GNG13), and Trpm5—in developing CVP by immunohistochemistry and/or *in situ* hybridization. We identified the appearance of cells expressing these TRCSMs (which are widely accepted as representative markers for differentiated taste cells) in serial sections from the posterior one-third of the embryonic tongue (Figure 1) [2],[6],[7],[10]. One series of sections from an entire CVP was subjected to each combination of antibodies or probes, such as PLCβ2 and IP3R3 antibodies, and more than five CVPs were subjected to histological analysis with each combination of markers at each developmental stage.

In previous studies, cytokeratin-8/Troma1 (CK8) staining revealed that the taste bud primordia in CVP appear from E15 onward during early development of the tongue epithelium in mice [18]; this is before the morphology of taste buds becomes evident, approximately 2 d postnatal (P2). Although a search was carried out for TRCSMs within the mouse tongue epithelium from E11 to E16, none were detected; TRCSM-positive cells appear as single isolated cells among the cell population immunoreactive against CK8 at E17 (Figure S1 and Table S1). These results indicate that TRCSM-positive cells appear in the developing CVP just before birth in mice (Figure 1, S1 and S2). Around the time of birth (E18 to P0), two or three TRCSM-positive cells were observable within the entire CVP (Figures 1, 4 and S2). These cells were not considered to be fully differentiated taste cells because they lacked certain crucial taste cell markers such as taste cell receptors. While previous studies reported incomplete overlapping of five TRCSMs in taste buds in the CVP of adult mice [19]–[21], we detected 100% colocalization of these TRCSMs (PLCβ2, gustducin, IP3R3, Ggamma13, and Trpm5) in the developing tongue epithelium, from E17 to at least P5 (Figures 1, S2 and Table S1). These results suggest that TRCSMs are expressed simultaneously in the same cell population during early development of the taste cell lineage in CVP.

### Computational Analysis of the Promoter Regions of TRCSMs

**Figure 2:**
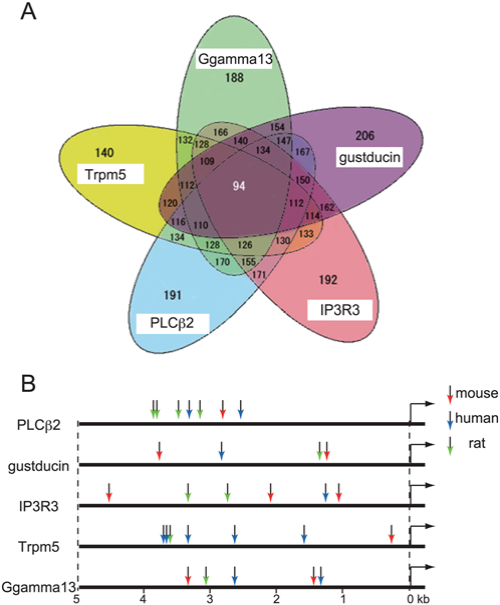
*In silico* analysis of the upstream region of TRCSMs in mammals. (A) Venn diagram representing the results of *in silico* analysis of the 5 kb upstream of TRCSM genes, including *Plcβ2*, *gustducin*, *Ip3r3*, *Trpm5*, and *Ggamma13* in mouse, human, and rat. Ninety-four transcription factors were identified as putative transcription regulators. (B) Summary of the putative HES1 binding sites in the 5 kb upstream sequence of each TRCSM. The putative binding sites on the mouse, human, and rat sequences are indicated with differently colored arrows (mouse, red; human, blue; and rat, green) on the horizontal lines, which represent the 5 kb upstream sequences of the TRCSMs.

The synchronous cellular colocalization of TRCSMs led us to investigate the regulatory mechanisms of TRCSMs, under the hypothesis that these genes are involved in the same regulatory network and share common regulatory factors, at least in the early phase of taste cell development. We analyzed the promoters of the five TRCSMs *in silico* to identify any common transcription factors that bind to regulatory sequences of taste stimuli signaling components. A series of putative transcription factor binding sites to these DNA sequences were identified by the Match program [22], which searched for regulatory sequences up to 5 kb upstream of each of the five TRCSMs (Figure 2). We further sieved common transcription factors through interspecies comparisons based on information acquired from mouse, rat, and human DNA sequences. Using these computational predictions, we identified 94 transcription factors as putative common transcription regulators (Figure 2A). These factors, which included candidates for factors implicated in the taste developmental system, are listed in Table S2. To evaluate this approach, we further performed a bibliographic and database search for gene expression within the embryonic oral epithelium. Because transcription repressors are presumably required to suppress the expression of TRCSMs in stem or precursor cells, we focused on transcription repressors within our list of identified candidates, in an effort to identify the regulator for taste stem cells or precursor cells. Ultimately, *Hes1*, a basic helix-loop-helix type of transcription factor, emerged as the most likely candidate from our different sets of informatics screenings (Figure S3).

### HES1 Binds the Promoters of *Plcβ2* and *Ip3r3*

**Figure 3:**
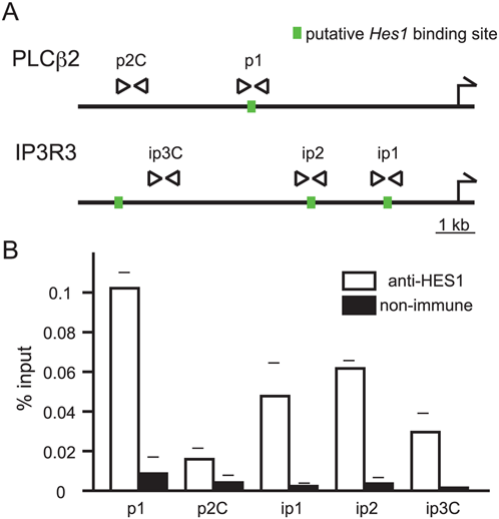
Binding of HES1 to TRCSM promoter sequences. (A) Position of HES1 binding sites within the promoter regions of *Plcβ2* and *Ip3r3* are indicated by green squares. Arrowheads indicate primer pairs used for ChIP assays. Primer pairs p1, ip1, and ip2 amplified the DNA fragment that included HES1 binding sites, while fragments amplified by p2C and ip3C primer sets did not contain the HES1 binding sequence. (B) ChIP results using P19 embryonal carcinoma cells as chromatin substrate. HES1 antibody efficiently precipitated sequences containing *Plcβ2* and *Ip3r3* promoter HES1 binding sites.

To confirm that HES1 binds to the *Plcβ2* and *Ip3r3* promoter regions (Figure 2B), we ran chromatin immunoprecipitation assays (ChIP) using an antibody against HES1. We designed several pairs of primers to amplify putative HES1 binding sites in these promoter regions, as predicted by our *in silico* analyses. As controls, we also designed pairs of primers that did not contain the HES1 binding sequence (Figure 3). ChIP with the p1 primer pair yielded a higher recovery of chromatin than did ChIP with the control p2C primer pair (Figure 3). Similarly, the ip1 and ip2 primer pairs also yielded a higher recovery of chromatin than did the control ip3C primer pair. The ip3C control primers showed a relatively high recovery of chromatin, most likely due to the close location to the third HES1 binding site, and to their position between two HES1 binding sites within the *Ip3r3* promoter region (Figure 3). These results suggest that HES1 bound the predicted sequences in the *Plcβ2* and *Ip3r3* promoter regions.

### Expression Analysis of *Hes1* in CVP

**Figure 4:**
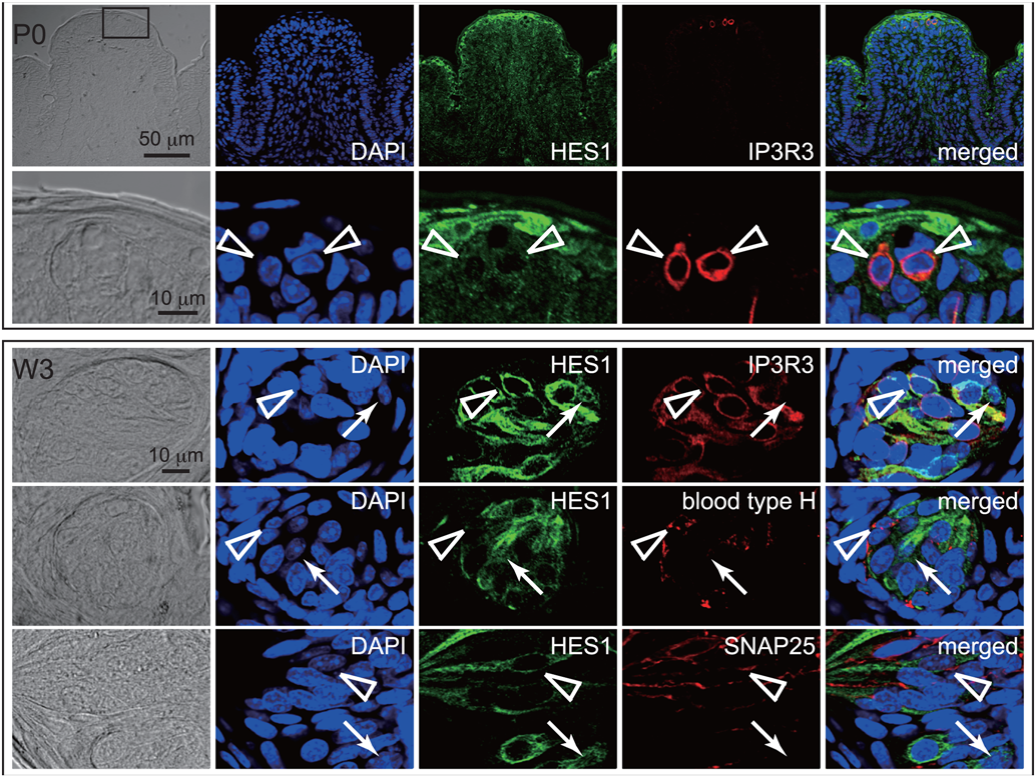
Immunohistochemical analysis of HES1 and IP3R3 in developing CVP at P0 and W3. HES1 immunoreactivity exhibited rather uniform distribution in CVP from P0 animals, whereas a few nuclei showed reduction of HES1 immunoreactivity. The cells with reduced nuclear HES1 immunoreactivity exhibited IP3R3 expression at P0. In W3 animals, most of the cells in the taste buds displayed cytoplasmic localization of HES1, suggesting that it was nonfunctional as a transcription regulator. The very few cells retaining HES1 in the nucleus are indicated by white arrows; these cells did not express IP3R3 or SNAP25. However, some of the cells with reduced HES1 reactivity in the nucleus expressed IP3R3, SNAP25, or blood type H antigen (arrowheads).

Because our composite approach to identifying factors in the regulatory network of taste system development picked up *Hes1* as a strong candidate, we further analyzed the role of *Hes1* in taste system development. *In situ* hybridization analyses against tongue epithelium from 3-weeks-old animals (W3) revealed that large numbers of cells within taste buds exhibited *Hes1* transcript (Figure S4) [23], and that expression of *Hes1* overlapped with the TRCSMs (data not shown). This observation contradicts somewhat the hypothesis that HES1 directly represses the expression of TRCSMs in taste buds; therefore, we performed detailed immunohistochemical analyses using HES1 antibody on CVPs from P0 animals and W3 animals (Figure 4). The TRCSM-positive cells observed at P0 showed a reduction in HES1 immunoreactivity within nucleus, suggesting that HES1 protein had evacuated from nuclei (Figure 4). In W3 animals, HES1 localized in the cytoplasm of most taste bud cells (Figure 4). This cytoplasmic HES1 can be considered to be nonfunctional as a transcription regulator. The few cells that showed HES1 localized in the nucleus as well as in the cytoplasm exhibited no IP3R3 expression (Figure 4; indicated by white arrows), while the cells with cytoplasmic HES1 only also expressed IP3R3 (Figure 4; indicated by arrowheads). This suggested that regulation of the subcellular localization of HES1 was important for taste cell differentiation.

Because HES1 represses transcription from bound promoters, cells positive for HES1 within the nucleus may be either precursor cells (including stem cells) of TRCSM-positive cells (type II; responsible for sweet, bitter, and umami taste) or other cell types within the taste cell lineage, such as type I or type III cells [10],[11]. Therefore, it is important to observe HES1 colocalization with markers for other differentiated cell types within taste buds [12],[24]. Similar results in the case of IP3R3 (Figure 4) were obtained with the blood type H antigen and SNAP25, which represent type I and type III cells, respectively, within taste buds (Figure 4) [12],[24]. Our results raise the possibility that HES1 is commonly expressed in precursor cells involved in the cell type differentiation pathway within CVP, and that *Hes1* activity is required in the precursor or stem cell population in taste system development.

### Loss of *Hes1* Activity Leads to Overexpression of TRCSMs in Developing CVP

**Figure 5:**
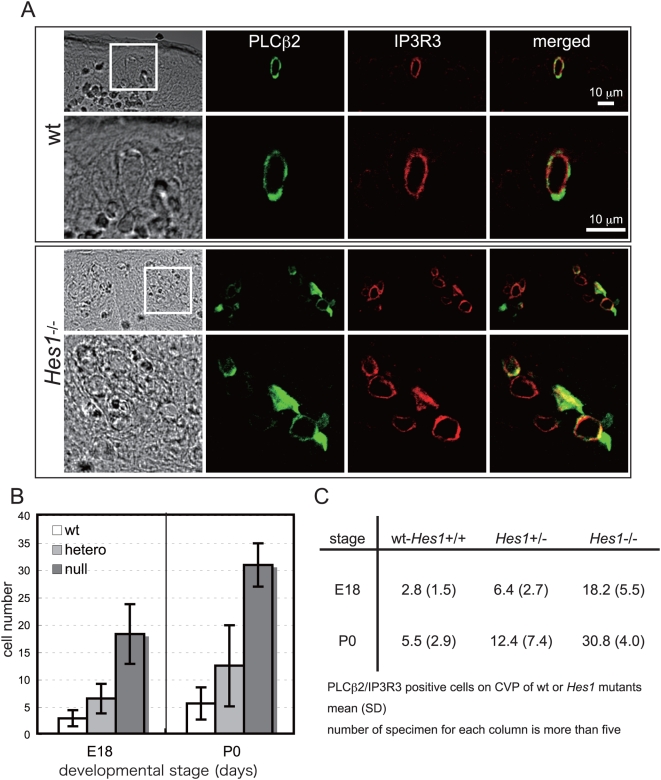
Gene dosage effect of *Hes1* on taste cell differentiation. (A) CVP from E18 embryos of wild-type and *Hes1* ^−*/*−^ mutant littermates were stained with antibodies against PLCβ2 and IP3R3. The developing taste buds from the oral epithelium of *Hes1* ^−*/*−^ mutants (lower panels) exhibited many more PLCβ2 (green) and/or IP3R3 (red) immunoreactive cells than did their wild-type littermates (upper panels), which displayed very few PLCβ2 and IP3R3 immunoreactive cells at this stage. Scale bar, 10 µm. (B) PLCβ2 and IP3R3 immunoreactive cells in *Hes1* mutant CVP at E18 and P0. Serial sections of entire CVP from wild-type, *Hes1* ^+/−^, and *Hes1* ^−*/*−^ littermates were immunostained with the PLCβ2 and IP3R3 antibodies, and immunoreactive cells were counted. The results represent the mean of more than five specimens. (C) The table shows the average and standard deviation (S.D.) of PLCβ2 and IP3R3 immunoreactive cells at E18 and P0 obtained from sections of entire CVP from wild-type, *Hes1* ^+/−^, and *Hes1* ^−*/*−^ littermates. The table is graphically displayed in Figure 5B. More than five specimens of each genotype and stage were used for counting the cells.

To clarify the potential role of *Hes1* during development of the taste recognition system *in vivo*, we performed analyses of taste cell differentiation in mouse *Hes1* mutants. Because *Hes1* ^−*/*−^ mice die at the newborn stage, observations of entire CVP by serial section were conducted around the time of birth. In wild-type littermates, PLCβ2/IP3R3-positive cells appeared as single, isolated cells (Figure 5A). However, in *Hes1* ^−*/*−^ embryos, the PLCβ2/IP3R3-positive cells were relatively small in shape, increased in number, and in contact with one another, forming cell clusters within the CVP of E18 embryos (Figure 5A). The total number of PLCβ2- and/or IP3R3-positive taste cells in the entire CVP was more than 5-fold greater in *Hes1* ^−*/*−^ embryos than in their wild-type littermates at E18 and P0 (Figure 5).

**Figure 6:**
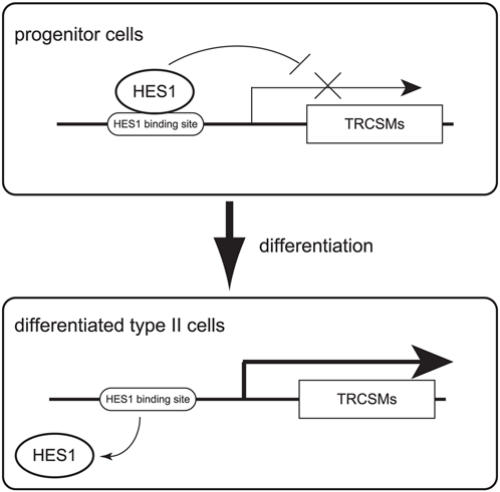
Schematic illustration of the transcriptional regulation of TRCSMs during differentiation of taste cells. HES1 activity is required to maintain the undifferentiated state and to repress the transcription of TRCSMs in developing immature taste cells. Loss of *Hes1* is accompanied by differentiation of taste cells expressing TRCSMs such as PLCβ2 and IP3R3.

Previous lineage tracing studies have indicated that taste cells are derived from as-yet unidentified stem cells that reside outside of taste buds, and that immature but postmitotic progenitors derived from these stem cells enter taste buds before the last division and final round of differentiation step [8]–[11]. Thus, the HES1 that we observed in cells within the taste buds (Figure 4) suggests that it may play a role in repressing TRCSMs in these progenitor cells (Figure 6). These observations support our hypothesis that *Hes1* functions as a repressor of TRCSMs in taste cell precursor cells.

## Discussion

Despite its importance, research regarding the molecular mechanisms of the development of the taste system has lagged behind that of the other special sense organs [3]–[5]. In our investigation of the molecular regulation of taste cell differentiation, we isolated key regulators of taste cell differentiation in early development by combining computational and experimental biology.

Sharing gene expression regulatory components is an efficient way of regulating molecules within the same signal transduction pathway. TRCSMs are indeed expressed in the same population of cells, at least during the course of early taste system development. We performed *in silico* analysis of stretches of sequence up to 5 kb upstream of TCRSMs, some of which had been shown previously to drive TCRSM expression in taste cells in transgenic mice [25],[26]. Using computational analysis to determine which transcription factor binding sites were commonly found in the promoters of genes involved in the same regulatory network, we identified a number of putative transcription regulators. Similar procedures could be applied to analyses of other systems.

It has been proposed that the development of taste buds is regulated by epithelial-mesenchymal interactions involving several different signaling pathways, such as Notch, Shh, Wnt, and BMP [23], [27]–[32]. Recent analyses of β-catenin and *Sox2* suggest that they are involved in taste cell development, although the steps involved in differentiation have yet to be clarified [27]–[29]. The expression patterns of Notch signaling pathway–related genes indicate that the Notch signaling cascade may have a role during morphological differentiation of CVP [23]. Here, we report that the number of TRCSM-positive cells is more than 5-fold greater in *Hes1* ^−*/*−^ embryos than in their wild-type littermates at stages E18 and P0 (Figure 5). Although we believe that the increase in TRCSM-positive cells observed in *Hes1* ^−*/*−^ mutants is due to premature expression of these marker proteins in the taste cell lineage, we cannot not exclude other possibilities, such as an increase in the total number of cells in CVP, or ectopic expression in cell types other than taste cells, in which expression of TRCSMs is normally repressed by HES1. Previous studies have proposed that a precursor population in the developing central nervous and hematopoietic systems expresses *Hes1* to maintain its undifferentiated state, and that downregulation of *Hes1* leads to differentiation [33]–[37]. *Hes1* may have a similar function in the taste cell lineage, and a reduction in nuclear HES1 would thus trigger taste cell differentiation in CVP epithelium. In addition, we observed a reduction in nuclear HES1 in blood type H antigen– and SNAP25-positive cells (corresponding to type I and type III taste bud cells, respectively) in older animals (Figure 4). These observations support the possibility that *Hes1* is indeed a common regulator of taste bud cell differentiation.

Our computational analysis yielded several transcription factors that may be involved in the TRCSM regulatory network (Table S2). Our investigation of HES1, one of the candidate transcription factors, provides support for the utility of the computational approach. Our list of TRCSM regulators will be a valuable resource for future studies of taste development, leading to a better understanding of the process of taste cell differentiation. Further, it may be useful for designing therapies for taste disorders, such as loss of taste.

## Materials and Methods

### Animals and Tissues

*Hes1* mutant animals were kindly provided by Ryoichiro Kageyama [37]. Developing CVP were fixed with neutralized 10% formaldehyde and embedded in paraffin. The histological protocols were described previously [38],[39]. The sections were 7.5 µm thick. Serial sections were prepared from the tongue, including entire CVP. A series of serial sections was subjected to immunohistochemistry or *in situ* hybridization with each combination of antibodies or probes (PLCβ2 and IP3R3, gustducin and IP3R3, *Plcβ2* and *Ggamma13*, and *Plcβ2* and *Trpm5*). For each combination of antibodies or probes, more than five serial section series were used for staining. Overall, tongues from more than 80 animals (four combinations of markers at stages E16, E17, P0, and P5) were analyzed to observe colocalization of TRCSMs (Figure 1).

### *In Silico* Analyses of Promoter Sequences

The mouse, rat, and human sequences 5 kb upstream of the TRCSMs that we investigated were retrieved from the Ensembl v46 (Aug 2007) database: *Plcβ2* (ENSMUST00000077829), *Trpm5* (ENSMUST0000009390), *gustducin* (*Gnat3*) (ENSMUST000000030561), *Ip3r3* (ENSMUST00000049308), and *Ggamma13* (*Gng13*) (ENSMUST00000026836). We utilized vertebrate-specific profiles of transcription factor binding sites (TFBSs) in the TRANSFAC Professional database 11.3 (10 September 2007). We searched for putative TFBSs in the promoter sequences of five TRCSMs in mouse, rat, and human using the MATCH program (version 10.4) [22], with the option of minimizing the number of the error rates of false positives and false negatives. Among the putative TFBSs that we discovered in these cross-species searches, we identified putative TFBSs that were conserved among all three species. We also performed a database search for genes expressed during stages E16–E18 in mouse undifferentiated oral epithelium in the Mouse Genome Informatics page of Jackson laboratory (as salivary gland precursor cells and oral epithelium at Theiler's Stage (TS) 24–TS26) (http://www.informatics.jax.org/) (Figure S3).

### ChIP

ChIP experiments were performed in accordance with previous reports [40],[41] and a technical protocol established by the Farnham laboratory (http://genomecenter.ucdavis.edu/farnham/farnham/protocols/tissues.html). We used stage P19 embryonal carcinoma cells as a substrate for ChIP assays, and an antibody against HES1 (Chemicon, AB5702: antibody raised against a synthetic peptide).

The following primers were used: p1 pair, 5′-TGTTAGAACGCTGGAGTTCAAG-3′ and 5′-ATCAGGCTCAGCTTTCCCATG-3′; p2C pair, 5′-AAAGTCTCTCGGACACCCAGC-3′ and 5′-TCTTAGGCTGTGAGGCAGCTG-3′; ip1 pair, 5′-GAGCAGAATGAGATCCGCATC-3′ and 5′-ACTGGGTAGCTGCTGCTACAG-3′; ip2 pair, 5′-CTCATTGACACCTGGGAGGAG-3′ and 5′-GGAATCTACATCCCTCAGTGG-3′; and ip3C pair, 5′-GTTGGGTCCAGAGTCAGAGAC-3′ and 5′-CTCACCTTCTAGGATCTCAGG-3′.

### Immunostaining

We used antibodies to PLCβ2 (Santa Cruz, SC206: antibody raised against amino acids 1170–1181 of PLCβ2 of human origin), gustducin (Santa Cruz, SC395: antibody raised against amino acids 93–112 of gustducin of rat origin), IP3R3 (BD Transduction Laboratories, 610312: antibody raised against amino acids 22–230 of IP3R3 of human origin), SNAP25 (Abcam, ab24737: antibody raised against full length protein-the critical epitope lies amino-terminal of the C-terminal peptide), and human blood type H antigen (AbH) (Abcam, ab3355: antibody raised against human colon cancer cell line SW-403). An antibody against HES1 was raised in this study using a polypeptide corresponding to amino acids 24–41 of HES1 (TPDKPKTASEHRKSSKPI) to immunize a rabbit and produce anti-HES1 antibody (Figure S5). Antiserum was purified by the same polypeptide. We verified the specificity of this antibody by western blotting and immunohistochemistry on the spinal cords of wild-type and *Hes1* ^−*/*−^ embryos (Figure S5). Our anti-HES1 antibody recognized nuclear localized HES1 in neurons from the embryonic spinal cord in wild-type animals (Figure S5).

All sections were treated with HistoVT One solution (Nakalai Tesque, 06380–05) for antigen retrieval. Images were captured with LSM51 confocal microscopy (Zeiss), and their optical thicknesses are 1 µm [42].

### Estimation of Immunoreactive Cells

All PLCβ2-, gustducin-, and IP3R3-positive cells were counted in 7.5 µm serial immunohistological sections from whole CVP. Immunoreactive cells were counted only when nuclear staining with DAPI was clearly observed in the same cell. Immunofluorescence that appeared at a similar position in two successive sections was counted as one positive cell. All immunoreactive cells were observed with an LSM51 confocal microscope (Zeiss) and the optical thicknesses of images are 1 µm [42].

## Supporting Information

Figure S1 Expression of PLCβ2 and CK8 in CVP epithelium at E17. Double color immunohistochemistry against CK8 (green) and PLCβ2 (red) in CVP at E17 revealed appearance of PLCβ2 positive cells within CK8 positive cell population.
(0.1 MB PDF)

Figure S2 Colocalization of TRCSMs in developing CVP. *Plcβ2*/*Ggamma13* or *Trpm5* expression in developing taste buds in the CVP from stages E17 to P5 was examined by double-color fluorescent *in situ* hybridization. *Plcβ2* (green) and *Ggamma13* or *Trpm5* (red) signals always colocalized in the same cells, at least until P5. Scale bar, 10 µm.
(0.3 MB PDF)

Figure S3 Experimental strategy for *in silico* analysis to identify putative common regulatory factors of TRCSMs.The flowchart indicates an experimental strategy of using *in silico* analysis to identify the putative common regulatory factors of TRCSMs.
(0.01 MB PDF)

Figure S4 *In situ* hybridization of *Hes1* in CVP epithelium. *Hes1* expression was stronger in the deep trench epithelial cells during early development of CVP (E17 and P0). In adults (10 wk after birth, W10), cells in taste buds strongly expressed *Hes1*. The rectangle in the third image indicates the field shown in the right-most image. Scale bars for the left three images, 100 µm. Scale bar for the right-most image, 50 µm.
(0.05 MB PDF)

Figure S5 Evaluation of the anti-HES1 antibody. We evaluated our anti-HES1 antibody by Western blotting and immunohistochemistry. Western blotting was carried out against a lysate of cos7 cells with a pCMV expression vector DNA without insert (mock, lane 1) and a DNA construct expressing a FLAG-HES1 fusion protein under the control of the CMV promoter (lane 2). The filter on the left was incubated with anti-HES1 antibody, and the filter on the right was incubated with anti-FLAG antibody (Sigma). The same band at about 30 kDa reacted against the antibody, suggesting that the band corresponded to the FLAG-HES1 fusion protein. Immunohistochemical tests were also carried out with *Hes1* ^−*/*−^ mutant and wild-type siblings. The immunohistochemistry of a spinal cord around floor plate from a wild-type embryo exhibited fluorescent signals in the nucleus, in the same pattern as with *in situ* hybridization signals. However, no obvious signals were observed from the spinal cords of *Hes1* ^−*/*−^ mutants. This suggests that the anti-HES1 antibody we raised exhibits HES1-specific immunoreactivity.
(0.09 MB PDF)

Table S1 TRCSM-positive cells in CVP at E17. We counted the cells positive for TRCSMs in early developing CVP from E17 embryos. The table indicates the number of CMVs subjected to two-color fluorescent histological analysis with each combination of markers, the total number of cells positive for each TRCSM tested, and the number of overlapping signals from a combination of two TRCSMs.
(0.04 MB PDF)

Table S2 Transcription factors identified by *in silico* analyses. Ninety-four transcription factors were identified by *in silico* analyses to be likely members of the TRCSM regulatory network.
(0.03MB PDF)
