# Supplementary figures and images for: Combined In Silico and In Vivo Analyses Reveal Role of Hes1 in Taste Cell Differentiation
Source: PLoS Genet. 2009 Apr 3;5(4):e1000443. doi: 10.1371/journal.pgen.1000443 (PMC2655725; doi:10.1371/journal.pgen.1000443)

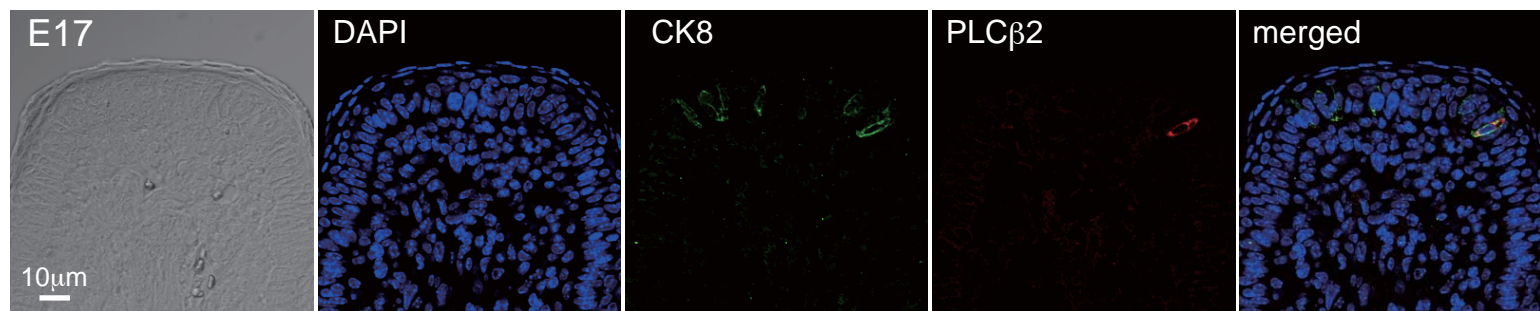

Supplement: Figure S1 — Expression of PLCβ2 and CK8 in CVP epithelium at E17. Double color immunohistochemistry against CK8 (green) and PLCβ2 (red) in CVP at E17 revealed appearance of PLCβ2 positive cells within CK8 positive cell population. (0.1 MB PDF) [file pgen.1000443.s001.pdf]

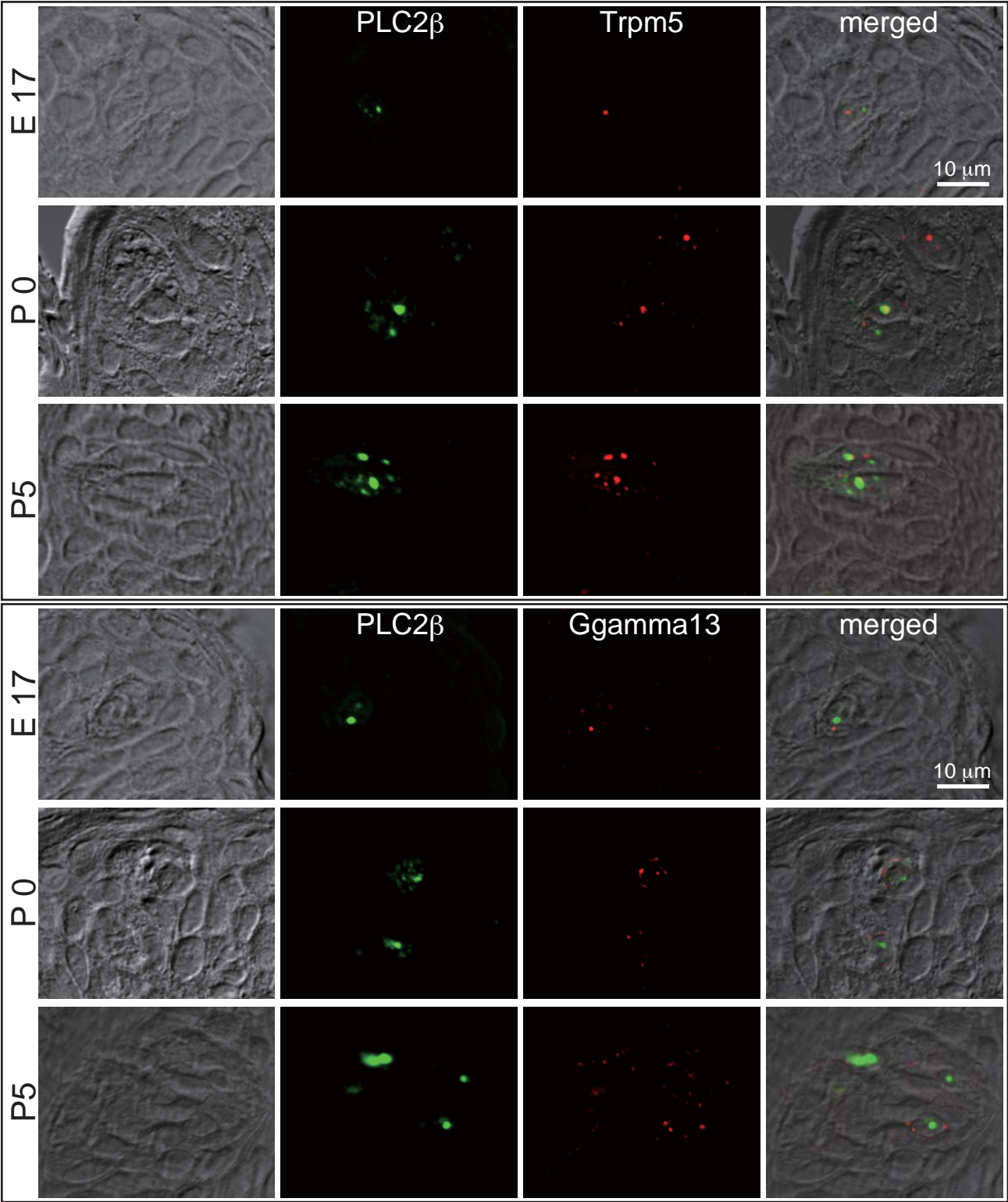

Supplement: Figure S2 — Colocalization of TRCSMs in developing CVP. Plcβ2/Ggamma13 or Trpm5 expression in developing taste buds in the CVP from stages E17 to P5 was examined by double-color fluorescent in situ hybridization. Plcβ2 (green) and Ggamma13 or Trpm5 (red) signals always colocalized in the same cells, at least until P5. Scale bar, 10 µm. (0.3 MB PDF) [file pgen.1000443.s002.pdf]

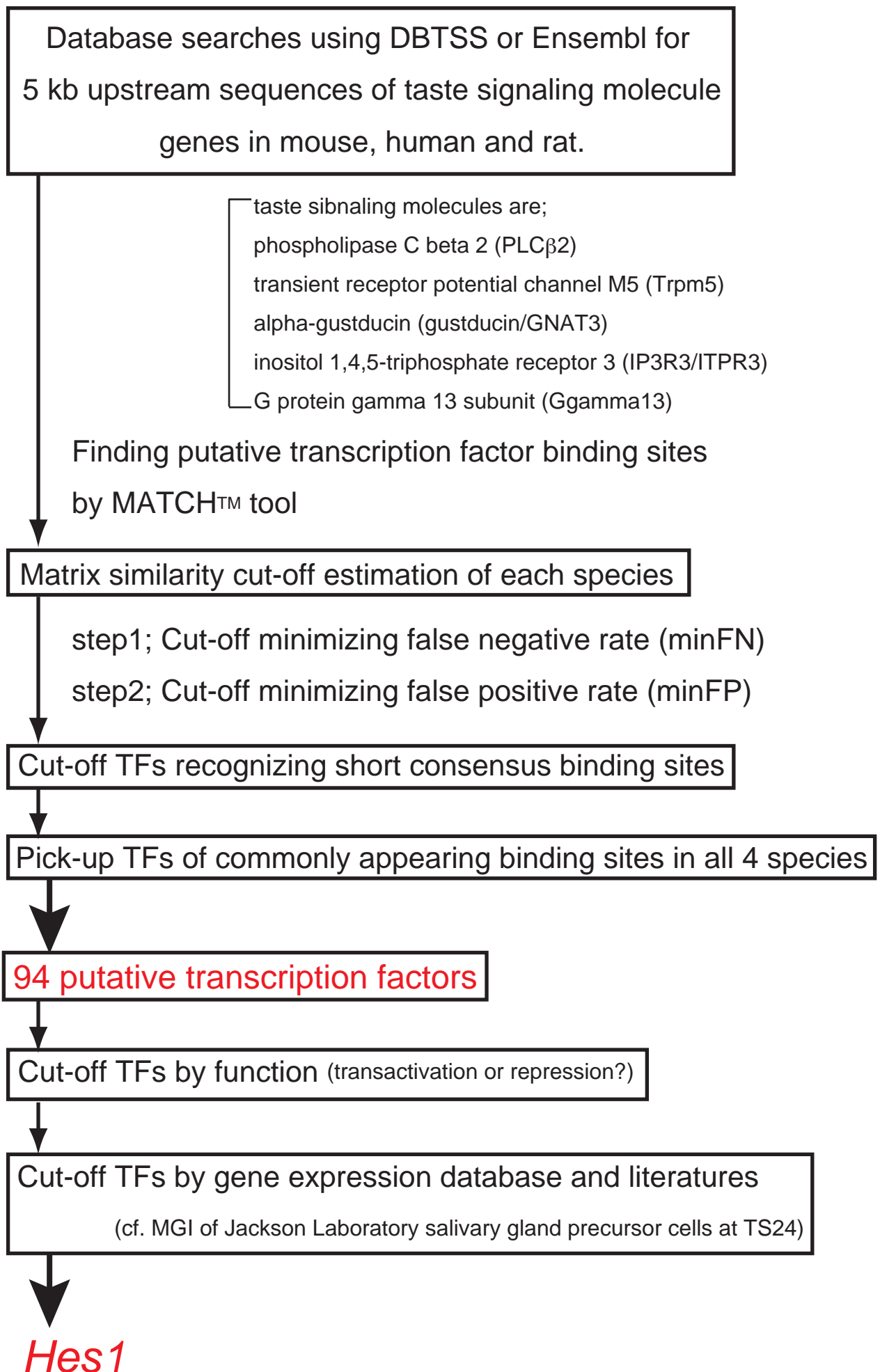

Supplement: Figure S3 — Experimental strategy for in silico analysis to identify putative common regulatory factors of TRCSMs.The flowchart indicates an experimental strategy of using in silico analysis to identify the putative common regulatory factors of TRCSMs. (0.01 MB PDF) [file pgen.1000443.s003.pdf]

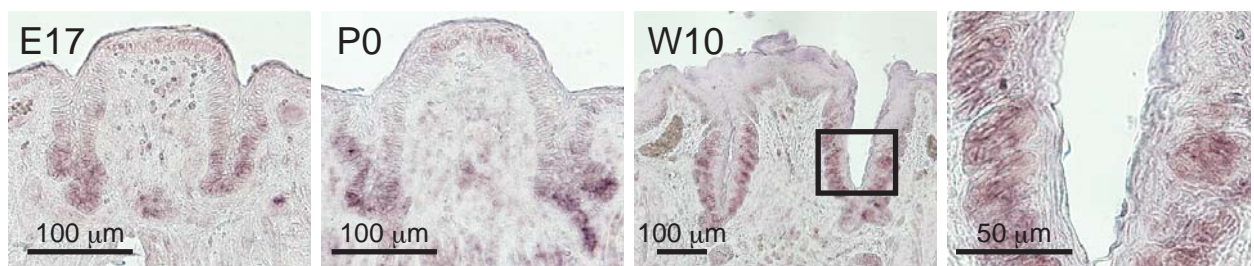

Supplement: Figure S4 — In situ hybridization of Hes1 in CVP epithelium. Hes1 expression was stronger in the deep trench epithelial cells during early development of CVP (E17 and P0). In adults (10 wk after birth, W10), cells in taste buds strongly expressed Hes1. The rectangle in the third image indicates the field shown in the right-most image. Scale bars for the left three images, 100 µm. Scale bar for the right-most image, 50 µm. (0.05 MB PDF) [file pgen.1000443.s004.pdf]

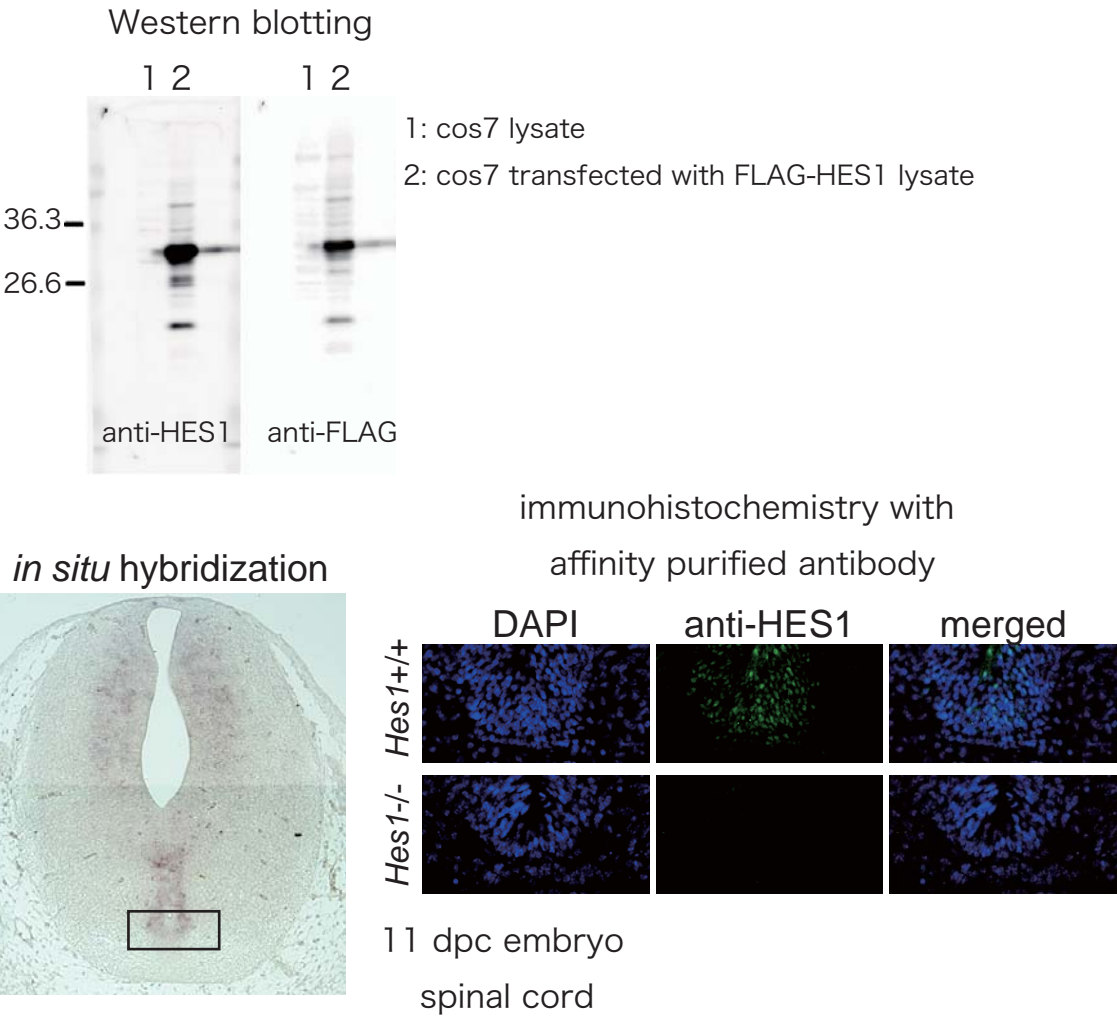

Supplement: Figure S5 — Evaluation of the anti-HES1 antibody. We evaluated our anti-HES1 antibody by Western blotting and immunohistochemistry. Western blotting was carried out against a lysate of cos7 cells with a pCMV expression vector DNA without insert (mock, lane 1) and a DNA construct expressing a FLAG-HES1 fusion protein under the control of the CMV promoter (lane 2). The filter on the left was incubated with anti-HES1 antibody, and the filter on the right was incubated with anti-FLAG antibody (Sigma). The same band at about 30 kDa reacted against the antibody, suggesting that the band corresponded to the FLAG-HES1 fusion protein. Immunohistochemical tests were also carried out with Hes1 −/− mutant and wild-type siblings. The immunohistochemistry of a spinal cord around floor plate from a wild-type embryo exhibited fluorescent signals in the nucleus, in the same pattern as with in situ hybridization signals. However, no obvious signals were observed from the spinal cords of Hes1 −/− mutants. This suggests that the anti-HES1 antibody we raised exhibits HES1-specific immunoreactivity. (0.09 MB PDF) [file pgen.1000443.s005.pdf]
