# Supplementary material for: Combined In Silico and In Vivo Analyses Reveal Role of Hes1 in Taste Cell Differentiation
Source: PLoS Genet. 2009 Apr 3;5(4):e1000443. doi: 10.1371/journal.pgen.1000443 (PMC2655725; doi:10.1371/journal.pgen.1000443)
Supplement: Table S1 — TRCSM-positive cells in CVP at E17. We counted the cells positive for TRCSMs in early developing CVP from E17 embryos. The table indicates the number of CMVs subjected to two-color fluorescent histological analysis with each combination of markers, the total number of cells positive for each TRCSM tested, and the number of overlapping signals from a combination of two TRCSMs. (0.04 MB PDF) [file pgen.1000443.s006.pdf]

Table S1. TRCSM-positive cells counted in CVP at E17

marker combination	number of CVP[1]	single TRCSM positive cells[2]	double TRCSMs positive cells[3]	overlapping of two TRCSMs (%)
PLCβ2/IP3R3	7	4 (0.57)	4 (0.57)	100
GUSTDUCIN/IP3R3	5	3 (0.6)	3 (0.6)	100
Plcβ2/Ggamma13	5	3 (0.6)	3 (0.6)	100
Plcβ2/Trpm5	5	3 (0.6)	3 (0.6)	100

1 Total number of CVP analyzed.

2 Total number of cells which exhibited immunoreactivity against at least one of two markers.
Number in rectangles indicate average number of TRCSM positive cells per single CMV.

3 Total number of cells which exhibited immunoreactivity against both two markers.
