# Supplementary material for: Combined In Silico and In Vivo Analyses Reveal Role of Hes1 in Taste Cell Differentiation
Source: PLoS Genet. 2009 Apr 3;5(4):e1000443. doi: 10.1371/journal.pgen.1000443 (PMC2655725; doi:10.1371/journal.pgen.1000443)
Supplement: Table S2 — Transcription factors identified by in silico analyses. Ninety-four transcription factors were identified by in silico analyses to be likely members of the TRCSM regulatory network. (0.03MB PDF) [file pgen.1000443.s007.pdf]

Table S2.
The list of transcription factors identified by *in silico* analyses

94 genes

Gene families	Gene symbols
Aire	aire
Ap-1	ap-1
Ap-2	ap-2alpha
	ap-2alphaa
	ap-2gamma
	ap-2rep
AP-4	ap-4
Ar	ar
Areb	areb6
Arp	arp-1
Atf	atf2
	atf4
	atf6
Arnt	arnt
Brca1	brca1:usf2
Car	car
Cdp	cdp
C/ebp	c/ebpbeta
	c/ebpgamma
Coup	coup
Cp2	cp2/lbp-1c/lsf
E-box	e2a
	e47
Elf	elf-1
Elk	elk-1
Ets	c-ets-1 68
	c-ets-1 p54
	c-ets-2
Fac	fac1
Foxo	foxo1
Gabp	gabp
Gata	gata-1
	gata-2
	gata-3
	gata-4
	gata-6
Gr	gr
Hand	hand1:e47
	helios a
Hes	hes1
Hnf	hnf4alpha
	hnf4 direct repeat 1
Ipf	ipf1
Ik	ik-2
Lef1	lef1
Lxr	lxr
Lyf-1	lyf-1
Maf	c-maf
	v-maf
Meis	meis1a:hoxa9
Movo	movo-b
Msx	msx-1
Mi	muscle initiator
Myb	c-myb

MyoD	myod
Myogenin	myogenin
Nerf	nerf1a
Nf-y	nf-y
NFκB	nf-kappab
Nkx2.5	nkx2.5
Pax	pax-2
	pax-3
	pax-5
	pax-8
Pea	pea3
Ppar	pparalpha:rxralpha
Pu.1	pu.1
Pxr	pxr
P53	p53 decamer
Rar	rar
Rfx	rfx1
Rush	rush-1alpha
Smad	smad4
Sox	sox10
srebp	srebp-1
Stat	stat3
	stat5a
Sp3	sp3
Spz	spz1
Tata	tata
Tbx	tbx5
Tcf	tcf1
	tcf11
Ttf	ttf1
Tal	tal-1alpha:e47
	tal-1beta:e47
	tal-1beta:itf-2
Tax	tax/creb
Usf	usf
Vdr	vdr
Yy1	yy1
Zf	zf5
Zic	zic1
	zic3
